# High-Throughput Tear Proteomics via In-Capillary Digestion for Biomarker Discovery

**DOI:** 10.3390/ijms252212239

**Published:** 2024-11-14

**Authors:** James Xiao, Kyla Frenia, Kathleen C. Garwood, Jeremy Kimmel, Leanne T. Labriola

**Affiliations:** 1 Retina Department, Sewickley Eye Group, Sewickley, PA 15143, USA; jamesxiao039@gmail.com; 2 Department of Bioengineering, Swanson School of Engineering, University of Pittsburgh, Pittsburgh, PA 15260, USA; 3 Department of Decision and System Sciences, Saint Joseph’s University, Philadelphia, PA 19131, USA

**Keywords:** tear proteomics, tear biomarker, biomarker discovery, point-of-care testing, POCT, LC–MS/MS

## Abstract

Tear fluid has emerged as a valuable resource for biomarker discovery; however, the limited sample volume, the dynamic composition, and the variability introduced by collection methods all present significant challenges to the analysis and interpretation of the results. A majority of tear proteomic studies have utilized Schirmer strips for tear fluid collection; however, microcapillary collection can provide a superior collection method for proteomic studies when analysis procedures are optimized. We developed a novel, high-throughput in-capillary trypsin digestion workflow that requires as little as 0.5 μL of tear fluid for bottom–up shotgun proteomics. The use of a single microcentrifuge tube for both tear collection and sample processing simplifies sample handling and minimizes both the sample loss and experimental errors associated with sample transfers. This streamlined approach also reduces sample processing time to under 2 h before overnight trypsin digestion, compared to the 5–8 h required by the other methods. Our method uses liquid chromatography–tandem mass spectrometry (LC–MS/MS) to identify more proteins with greater efficiency than the existing techniques. With this workflow, we identified 500–800 proteins per 0.5 μL sample without peptide fractionation, allowing for at least three technical replicates. The results showed a four-fold increase in the number of proteins identified in the samples. This approach validates the use of microcapillary tear collection, and the innovative processing technique significantly increases the throughput of tear proteomics for biomarker discovery.

## 1. Introduction

Tear fluid holds great promise as a non-invasive source of biomarkers for both ophthalmic and systemic diseases [1,2,3,4,5,6,7,8,9,10]. Unlike other biofluids, such as blood and cerebrospinal fluid, the collection of tear fluid is non-invasive and causes little discomfort, making it ideal for real-time point-of-care testing (POCT) [11,12,13,14,15]. However, the limited volume of tear fluid presents challenges for proteomics research [16]. Moreover, common tear collection methods, such as Schirmer strips and cellulose sponges, often require anesthetic drops and lengthy collection times. This process can stimulate reflex tearing in response to mechanical eye stimulation, altering the sample composition and compromising the reliability of sampling and analysis [17,18]. These collection methods, which require contact with the eyelid, can also introduce contamination from eyelid cellular debris, complicating the interpretation of proteomic results [3,11,16,17,19,20,21]. As a result, tear fluid proteomics analyses can vary substantially based on the sample collection method.

Microcapillary tubes (capillary tubes) offer an alternative method for tear sample collection that significantly reduces reflex tearing, minimizes cellular contamination, and enhances patient comfort [11,16,22,23]. To collect tear fluid with a capillary tube, the tip of the glass tube is placed in the lower eyelid margin in the area where tear fluid collects called the tear lake. Using this approach, a capillary tube can collect volumes of 0.1 μL to 2 μL, with an average of 0.5 μL in under one minute. This method creates less ocular irritation and therefore less stimulation of reflex tearing during collection. In addition, capillary collection requires minimal contact with the skin, reducing cellular contamination and yielding data with a higher proportion of extracellular proteins [2,24,25]. Thus, capillary tubes provide an excellent option for tear collection.

However, despite these advantages, this method has not been widely adopted for proteomics analysis and biomarker discovery, in part due to the lack of protocols capable of yielding sufficient protein identification from the limited volumes collected. Existing protocols for processing capillary tube-collected tear samples typically require the pooling of 5–20 μL of tears, which can be impractical to obtain as the basal tear film averages 3–10 μL of tear fluid [26]. Obtaining higher volumes of tear fluid requires the flushing of the tear fluid, which distorts the characteristics of the basal tear fluid [8,27,28,29,30,31,32,33]. Methods for the processing of tear fluid samples collected using capillary tubes reported a typical yield of only 100–200 identifiable proteins—substantially less than other methods—even when analyzing large volume and pooled samples [8,27,28,29,30,31,32,33,34,35,36]. Given the limited optimization of LC–MS/MS for small-volume capillary samples, these results may reflect limitations in the processing rather than the actual protein content.

To fill this gap, we have developed a rapid and efficient in-capillary digestion workflow tailored specifically for small-volume tear fluid proteomics. This novel method requires only 0.5 μL of tear fluid and eliminates sample transfer between collection, processing, and analysis, thereby significantly reducing the risk of sample loss, contamination, and associated errors. Using LC–MS/MS, this new method simplifies experimental procedures, shortens the sample preparation and handling time, and consistently identifies a higher number of proteins in tear fluid samples compared to previously reported approaches, paving the way for advancements in real-time POCT, disease detection and management, and personalized medicine.

## 2. Results

### 2.1. Development of a Novel In-Capillary Digestion Workflow for Enhanced Tear Proteome in Biomarker Discovery

**Figure 1:**
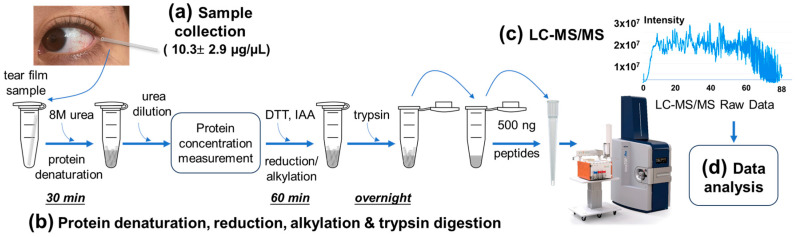
A novel in-capillary digestion workflow for enhanced tear proteomics in biomarker discovery. The LC–MS/MS workflow was optimized for low-volume tear samples. (**a**) Tear fluid samples, with an average protein concentration of 10.3 ± 2.9 µg/µL, were collected using 0.5 µL glass capillary tubes. (**b**) Experimental procedures for protein denaturation, reduction, alkylation, and trypsin digestion were conducted. To minimize potential sample loss and errors associated with transferring small-volume samples, 8 M urea solution was directly added to the microcentrifuge tube containing the capillary tube with 0.5 μL tear fluid, without flushing out the tear fluid sample. The tube was then rigorously vortexed and centrifuged at the maximum speed (~20,000× *g*) in a benchtop microcentrifuge to break the capillary tube into small pieces and thoroughly mix the tear samples with the urea solution. The sample was incubated at room temperature for 30 min to promote protein denaturation and then diluted fivefold to reduce the urea concentration to 1.6 M prior to protein quantification via A280 measurement on a NanoDrop spectrophotometer. Subsequently, proteins were reduced with dithiothreitol (DTT) at 56 °C for 30 min and alkylated with iodoacetamide (IAA) at room temperature in the dark before overnight trypsin digestion. After digestion and pH adjustment to 2–3, 500 ng of digested peptides were directly loaded onto an Evosep tip for LC–MS/MS analysis (**c**), followed by data analysis (**d**).

We developed a novel proteomic method designed for tear film fluid collection in microcapillary glass tubes to enhance LC–MS/MS protein identification (Figure 1). Tear samples were collected using 0.5 μL capillary tubes and transported to the laboratory on dry ice (Figure 1a). Instead of flushing the samples out of the tube, which can lead to sample dilution and loss, 8 M urea was added directly to the collection tube. This allowed for seamless protein solubilization and denaturation. Next, rigorous vortexing and high-speed centrifugation ensured the efficient extraction of tear fluid samples from the capillary and thorough mixing with the urea solution maximized the protein recovery (Figure 1b). This method both preserved the integrity of the tear sample and minimized the processing steps that usually contribute to inefficiencies in traditional workflows. Then, the peptide samples were loaded onto an Evosep tip using a modified protocol, including extensive washing steps to remove residual urea, before undergoing LC–MS/MS and data analysis (Figure 1c,d).

By optimizing these sample handling and processing steps, we were able to streamline the workflow, reducing the total sample preparation time (excluding the overnight trypsin digestion) to under 2 h. Notably, we eliminated the need for peptide fractionation and desalting, steps that are usually time-consuming and prone to sample loss. This represents a significant improvement over conventional protocols, which typically require 5–8 h (Figure S1) [10,19,21,36,37]. These optimizations translated into more rapid and reproducible analyses, improving the sensitivity and efficiency of tear proteomics for biomarker discovery in research and clinical applications.

### 2.2. Optimization of Protein Quantities and LC Gradients for LC-MS/MS Analysis

**Figure 2:**
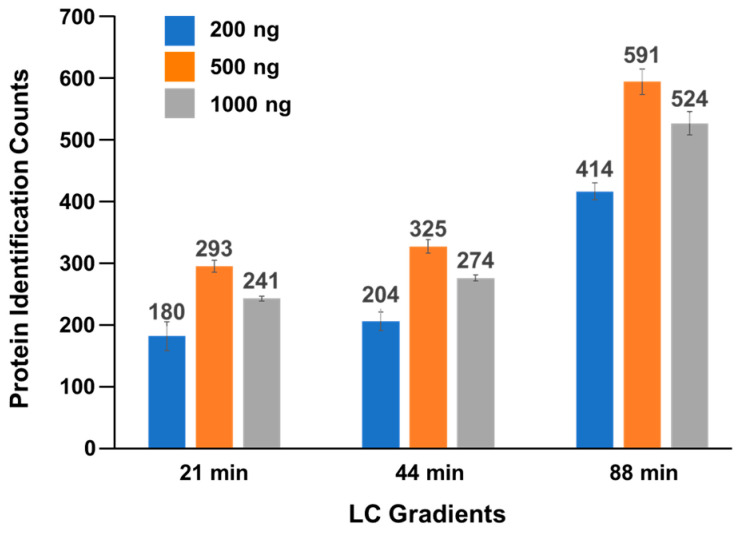
Determining optimal protein quantities and LC gradients for tear fluid samples. Three different amounts of tear protein peptides (200, 500, and 1000 ng) were tested using three different LC gradients (21, 44, and 88 mins). The experiments were repeated three times.

To determine the optimal amount of tear peptides for loading onto an Evosep tip for LC–MS/MS analysis, we prepared peptide solutions from the pooled tear samples collected from 12 individuals (6 males and 6 females, aged 43.6 (15–80) ± 23.6 years). Three different peptide amounts were tested—200 ng, 500 ng, and 1000 ng—using LC gradients of 21, 44, and 88 min (Figure 2). Each experiment was repeated in triplicate. The average number of proteins identified from each experiment ranged from 171 to 615. The highest number of identifications, 615 proteins, was observed using a loading mass of 500 ng and an 88 min gradient (Figure 2 and Table S1). Based on these results, 500 ng of peptides with the 88 min gradient were identified as the optimal condition for our tear proteomics method.

### 2.3. Urea Volume Optimization

To enhance protein denaturation in our tear proteomics workflow, we optimized the volume of 8 M urea used. In our initial workflow, 50 µL of 8 M urea was added to the tear samples to denature the proteins before digestion. As urea interferes with LC–MS/MS analysis, it must be removed during the later stages of sample processing. To streamline the workflow, we introduced an Evosep C18 tip wash step to remove urea, eliminating the need for additional desalting steps. Optimizing urea volume was crucial; too little would prevent thorough mixing with the tear fluid, while too much could lead to incomplete desalting, negatively affecting LC–MS/MS performance and reducing the number of proteins identified.

**Figure 3:**
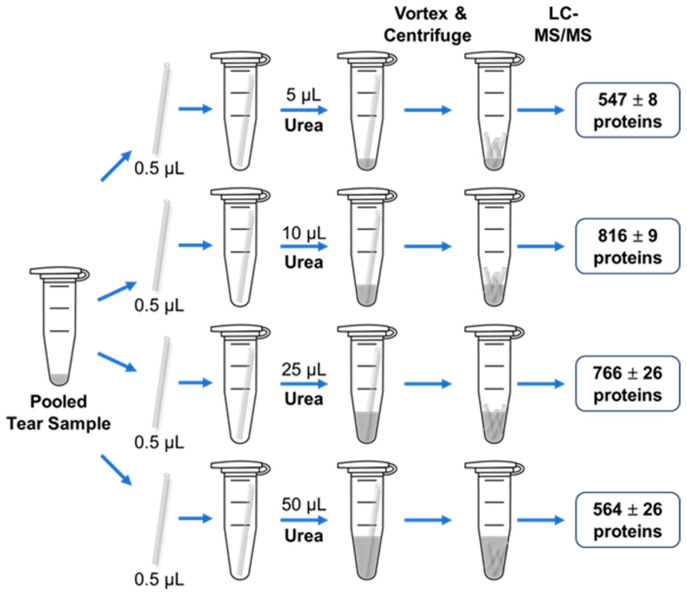
Optimization of 8 M urea solution volume. Pooled tear samples were collected in 0.5 µL glass capillary tubes and transferred to Protein LoBind microcentrifuge tubes. Different volumes of 8 M urea (5, 10, 25, and 50 µL) were added to each tube, followed by the workflow described in Figure 1. The tube with 10 µL of 8 M urea resulted in the highest number of identified tear proteins (816 ± 9 proteins). The experiments were repeated three times.

Four different volumes of 8 M urea, 5, 10, 25, and 50 µL, were tested following the procedures outlined in Figure 1. Testing was performed on the capillary tubes loaded with the pooled samples. The average number of tear proteins identified for each condition was 547 ± 8, 816 ± 9, 766 ± 26, and 564 ± 26, respectively (Figure 3 and Table S3). The results showed that 10 μL of 8 M urea yielded the highest number of protein identifications, making it the optimal volume for this workflow.

### 2.4. Protein Concentration Measurement Before DTT Reduction and IAA Alkylation

Accurate protein quantification is essential for translational and clinical quantitative proteomics, as well as for biomarker discovery. Due to the small volume of tear samples, direct protein concentration measurements on the original tear samples are not feasible. Instead, these measurements should be performed during sample processing once a sufficient volume is achieved by the addition of a buffer solution. Following buffer addition, there are several points in the workflow where the sample volume is sufficiently large enough for protein concentration measurement. However, some reagents in the buffer solution can interfere with protein measurements [38,39]. Sufficient volume is achieved during the following steps: (i) after denaturation when the urea concentration is diluted from 8 M to 1.6 M; (ii) after the addition of DTT for reduction; and (iii) after the addition of IAA for alkylation (Figure 1b). To determine the optimal step for protein measurements, we prepared solutions corresponding to these steps and evaluated the tear protein concentration measurements under varying reagent conditions. Solutions were prepared by mixing 0.5 μL aliquots of pooled sample with the respective buffer solutions (Figure S2a,b). Measurements were taken at 0, 5, 15, 30, 60, 90, and 120 min.

We used A280 absorbance measurements on a NanoDrop spectrophotometer, which requires only 1–2 µL of sample for protein quantification. Our results showed that tear protein concentration measurement remained stable even after 2 h at room temperature in the presence of 1.6 M urea. However, the addition of DTT and IAA significantly altered the A280 absorbance of the buffer solutions, which impacted the tear sample measurements. In addition, A280 absorbance changed with the longer incubation times in the presence of DTT and IAA (Figure S2a,b). These findings suggest that the most suitable step for A280 protein measurement is after the denaturation step, when the urea concentration has been diluted from 8 M to 1.6 M but before the addition of DTT and IAA (Figure 1a and Figure S1). This step provides the most reliable and stable measurements with minimal reagent interference, regardless of the timing of sample preparation. Using this method, we measured the protein concentrations of 22 individual tear samples, yielding an average protein concentration of 10.3 ± 2.9 µg/µL (Table S2).

During the tear protein concentration measurements described above, some samples were found to have a protein concentration of less than 10 µg/µL, resulting in A280 absorbance readings of below 0.1 on the NanoDrop spectrophotometer, which may increase measurement error. To address this, we further optimized the workflow. We reduced the volume of 50 mM ammonium bicarbonate buffer from 40 µL to 20 µL to improve the accuracy of the protein measurement. The protein concentration measurement was then performed in the presence of 2.67 M urea (Figure S2c,d). Following the measurement, an additional 20 µL of 50 mM ammonium bicarbonate was added to reduce the urea concentration to 1.6 M before proceeding with DTT reduction and IAA alkylation (Figure S1).

### 2.5. Tear Proteome and Tear Protein Functions

**Figure 4:**
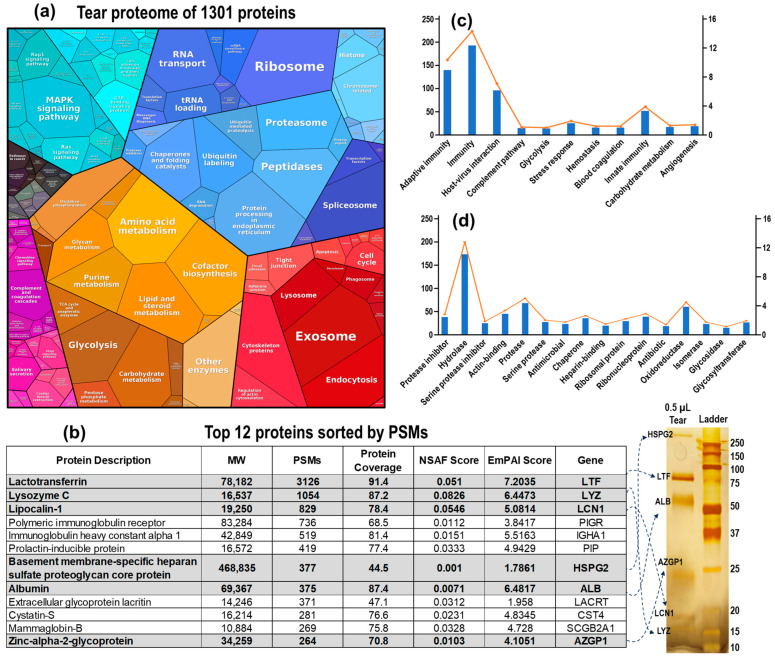
Overview of Tear Proteome—Protein Functions and Classifications. (**a**) Proteomap depicting the 1301 proteins identified in this study, collected using the glass capillary tube method. (**b**) Top 12 Tear Proteins ranked by PSMs (peptide-spectrum matches) in a representative tear proteomics dataset. Six of these proteins correspond to the prominent bands visible on the silver-stained SDS-PAGE gel from 0.5 µL of tear samples. (**c**,**d**) GO analysis of the 1301 tear proteins, revealing their involvement in biological processes (**c**) and molecular functions (**d**), was performed using DAVID Bioinformatics software (https://david.ncifcrf.gov/ accessed on 18 August 2024). Note: NSAS Score: Normalized Spectral Abundance Factor score; EmPAI Score: Exponentially Modified Protein Abundance Index score.

In this study, we collected a total of 39 LC–MS/MS datasets from tear samples; a total of 27 datasets were obtained from experiments focused on optimizing the loading amount and gradient, while 12 datasets were collected during experiments aimed at optimizing the volume of urea. By integrating these datasets, we identified a total of 1301 proteins in tear fluid (Figure 4a and Table S4). To gain a deeper insight into the tear proteome and its dynamic range, we performed SDS-PAGE analysis using 0.5 µL of tear sample, followed by an analysis with silver staining. The visible bands from the silver staining were excised, subjected to in-gel digestion, and then analyzed by LC–MS/MS (Figure 4b). We compared these results with the top 12 proteins from a representative dataset obtained using our in-capillary tear proteomics workflow. All visible bands on the silver-stained SDS-PAGE gel corresponded to the top 12 proteins among the 825 total identified proteins, which were ranked by peptide-spectrum matches (PSMs).

Next, we conducted Gene Ontology (GO) analysis to explore the biological processes and molecular functions of the identified proteins (Figure 4c,d and Table S5). The GO biological process analysis indicated that tear proteins are primarily involved in immunity, host–virus interaction, complementary pathways, and angiogenesis (Figure 4c). The GO molecular function analysis revealed that the tear proteome includes protease inhibitors, hydrolases, proteases, antimicrobial and antibiotic proteins, chaperones, and glycosidases (Figure 4d).

## 3. Materials and Methods

### 3.1. Tear Fluid Sample Collection and Processing

The study was approved by Advarra IRB (number Pro00072069), and written informed consent was obtained from all the individuals who participated. Research adhered to the tenets set forth in the Declaration of Helsinki and was conducted in accordance with regulations from the Health Insurance Portability and Accountability Act. Initially, a pooled tear sample was created in order to use a higher volume of tear with the same fluid composition as we focused on process optimization. To create a pooled tear sample, tear fluid was collected from 12 unique individuals, including 6 males and 6 females, with an average age of 43.6 (15–80) ± 23.6 years. We utilized the standard capillary tube tear collection method with Drummond™ Short-Length Microcaps™ Micropipettes (Fisher Scientific, Waltham, MA, USA) [2,22,40,41]. These capillary tubes are available from Fisher Scientific, and they offer a range of collection volumes from 0.5 µL to 20 µL. In our pilot testing, we found that the 0.5 µL capillary tubes (Catalog No. 22-249512, Fisher Scientific, Waltham, MA, USA)) typically yielded a full capillary in under one minute, effectively minimizing eye irritation and preventing reflex tearing that may occur with larger volumes collected over extended periods. Additionally, the 0.5 µL capillary tubes can be directly placed into 1.5 mL Protein LoBind microcentrifuge tubes without the need to flush out the tear samples, thereby simplifying the workflow. After tear collection, the capillaries containing fluid samples were placed into empty 1.5 mL Protein LoBind microcentrifuge tubes and frozen in a −20 °C freezer for short-time storage (several hours to overnight) in the eye clinics. The tear samples were transported to the research laboratory on dry ice and then stored in an −80 °C freezer before processing.

### 3.2. Initial Workflow for In-Capillary Digestion of Tear Fluid Samples

For pooled sample preparation, multiple capillaries containing 0.5 μL of tear film samples were combined in a single Protein LoBind microcentrifuge tube and centrifuged at 3000× *g* for 1 min. The tear samples were then vortexed thoroughly to ensure a uniform mixture before creating 0.5 μL aliquots for analysis using capillary tubes. Each capillary was placed in a 1.5 mL Protein LoBind microcentrifuge tube, and 49.5 μL of 8 M urea in 50 mM ammonium bicarbonate (Catalog No. 09830-500G, Millipore Sigma, Burlington, MA, USA) was added. The microcentrifuge tubes were vortexed vigorously and then centrifuged in the benchtop microcentrifuge at ~20,000× *g* for 5 min to break the capillary tube into small pieces and thoroughly mix the tear fluid samples with the urea solution. This procedure was repeated three times. The samples were then incubated at room temperature for 30 min to promote protein denaturation. Following this, the samples were diluted fivefold to reduce the urea concentration to 1.6 M prior to protein quantification. Protein concentrations of the tear samples were measured at A280 with a NanoDrop One Microvolume UV–Vis Spectrophotometer, using 1.6 M urea in 50 mM ammonium bicarbonate as a blank. Then, 1 μL of 500 μM DL-Dithiothreitol (DTT) (Catalog No. 3860-5GM, Millipore Sigma, Burlington, MA, USA) was added to the above solution to make a final DTT concentration of 10 mM. The samples were incubated at 56 °C for 30 min for reduction to occur. After reduction, iodoacetamide (IAA) (Millipore Sigma, Catalog No. I6125-5G) was added to the sample, resulting in a final concentration of 25 mM, followed by an incubation of 30 min at room temperature in the dark. Trypsin/Lys-C Mix, Mass Spec Grade (Catalog No. V5071, Promega, Madison, WI, USA), was added to the tear samples at a 1:20 protease-to-protein ratio. The samples were then digested for 16 h at 37 °C.

### 3.3. Optimization of Protein Quantities and LC Gradients for LC–MS/MS Analysis

Following digestion, the enzyme reaction was quenched by adding 3 µL of 50% formic acid, lowering the pH to 3–4. The tryptic peptide samples were then prepared in amounts of 200 ng, 500 ng, and 1000 ng, based on the protein concentrations measured in the earlier steps. The peptide samples were loaded onto Evosep tips (EV2011) following the manufacturer’s instructions prior to LC–MS/MS analysis. LC gradients of 21, 44, and 88 min were used for peptide separation.

### 3.4. Optimization of 8 M Urea Volume for Complete Trypsin Digestion of Tear Proteins and Maximizing Protein Identifications

Pooled tear samples were aliquoted into 0.5 μL capillary tubes and placed into 1.5 mL Protein LoBind microcentrifuge tubes. To each microcentrifuge tube, 10 μL of 8 M urea in 50 mM ammonium bicarbonate was added. To ensure thorough mixing of the small volume of tear samples with the urea solution, the microcentrifuge tubes were vortexed vigorously and then centrifuged in a benchtop microcentrifuge at ~20,000× *g* for 5 min, breaking the capillary tubes into small pieces and thus allowing the tear fluid to fully mix with the buffer solution. This procedure was repeated three times. The samples were then incubated at room temperature for 30 min to promote protein denaturation. Following this, 20 μL of 50 mM ammonium bicarbonate was added to dilute the urea concentration to 2.67 M and bring the total volume to 30 μL. Protein concentrations of the tear samples were measured at A280 using a NanoDrop One Microvolume UV–Vis Spectrophotometer, with the 2.67 M urea solution as a blank. An additional 20 μL of 50 mM ammonium bicarbonate was added, followed by 1 μL of 500 μM DTT (Catalog No. 3860-5GM, Millipore Sigma, Burlington, MA, USA) to achieve a final DTT concentration of 10 mM. The samples were incubated at 56 °C for 30 min for reduction to occur. After reduction, IAA (Millipore Sigma, Catalog No. I6125-5G) was added to the samples for a final concentration of 25 mM, and they were incubated for 30 min at room temperature in the dark. Finally, Trypsin/Lys-C Mix, Mass Spec Grade (Catalog No. V5071, Promega, Madison, WI, USA), was added to the tear samples at a 1:20 protease-to-protein ratio, and the samples were digested for 16 h at 37 °C.

### 3.5. Test the Effect of DTT and IAA on Tear Protein Concentration Measurements

The pooled tear samples were aliquoted into capillary tubes, each containing 0.5 µL of tear fluid, and then placed in individual Protein LoBind microcentrifuge tubes. Then, 10 µL of 8 M urea solution was added to each tube. Next, the samples were incubated at room temperature for 30 min to denature the proteins. Following this, either 40 µL or 20 µL of 50 mM ammonium bicarbonate solution was added to dilute the urea concentration. DTT and IAA were then added to the tubes as follows: S1 (no DTT or IAA), S2 (10 mM DTT), and S3 (10 mM DTT followed by 25 mM IAA). The volumes in all the tubes were adjusted to be equal by adding 50 mM ammonium bicarbonate solution. Corresponding buffer controls (B1, B2, and B3) were prepared without tear proteins to match the experimental conditions of the tear samples. Next, the tear protein and buffer solutions were incubated at room temperature, and A280 absorbance was measured at 0, 5, 15, 30, 45, 60, 90, and 120 min using a NanoDrop spectrophotometer.

### 3.6. LC–MS/MS Analysis

The MS data were collected with a timsTOF Pro 2 mass spectrometer coupled with an Evosep One LC system that was connected to an 8 cm Evosep performance column (EV-1109) using the 60 samples-per-day extended gradient. The tear peptide samples were analyzed with a standard data-dependent acquisition method, with parallel accumulation-serial fragmentation (DDA-PASEF), using the instrument in positive mode. A full scan was first collected with the mass range of 100–1700 *m*/*z* and the TIMS 1/k0 range of 0.60–1.60 V·s/cm^2^. During a full 1.17 s cycle, 10 PASEF ramps were performed, with the ramp and accumulation time both set to 100 ms. The following parameters were applied for all the DDA-PASEF HCD data collections: precursor intensity threshold: 2.5E3; charge state: +1 to +5; dynamic exclusion: 0.4 min; target intensity for fragmentation: 2E4; isolation window (linear): 2 *m*/*z* at 700 *m*/*z* and 3 *m*/*z* at 800 *m*/*z*; and collision energy (linear): 20 eV at1/k0 of 0.60 V·s/cm^2^ and 59 eV at 1/k0 of 1.60 V·s/cm^2^.

### 3.7. LC–MS/MS Database Search

All the DDA raw datasets were searched against the Uniprot reviewed human protein database (downloaded 30 March 2023) with BPS (Bruker ProteoScape) (ver. 2024). The trypsin activity was set to be fully specific (cleaves peptide bonds on the carboxyl side of the amino acids lysine (K) and arginine (R), except when either of these residues is followed by proline (P)), with one missed cleavage site allowed. A maximum of two of the following variable modifications were allowed on each peptide: C [+57.021464], STY [+79.966331], and M [+15.994915]. The precursor and the fragment mass tolerances were set to ±10 ppm and ±20 ppm, respectively. For protein identification, at least one associated peptide was identified within ±5 ppm of its theoretical *m*/*z* value. The Xcorr score cutoff at 1 was applied to +1 peptides, whereas for peptides with a higher charge state, the Xcorr score cutoff was set at 0.8, with the deltaCN cutoff set at 0.1 for all peptides. Additionally, only peptides with spectral evidence on at least 40% of the fragments (to aim for ≥80% sequence coverage) were considered for protein statistical calculations.

### 3.8. Silver Staining Analysis of Tear Samples

For silver staining, 0.5 μL of the tear film sample was mixed with 10 μL of 2× SDS-PAGE sample buffer and separated by SDS-PAGE electrophoresis. The gel was fixed in 10% methanol and 10% acetic acid for 30 min or overnight. The gel was then washed four times in water, with each wash lasting at least 5 min, taking care not to overwash it. Next, the gels were incubated in sodium thiosulfate (1 or 2 pellets, approximately 400 mg, per 500 mL of water) for exactly 90 s. A 20 mL aliquot of this sodium thiosulfate solution was reserved for the developing solution. After incubation, the gels were quickly washed three times with water. The gels were then immersed in a silver nitrate solution (0.9 g of silver nitrate in 500 mL of water) and allowed to stain for 10 min until they turned slightly yellow. The silver nitrate solution can be recovered and reused. After staining, the gels were washed quickly three times with water. Finally, the developer solution was prepared by mixing 10 g of potassium carbonate, 20 mL of the sodium thiosulfate solution from the previous step, and 250 µL of 40% formaldehyde in 500 mL of water. The reaction was stopped by adding a destaining solution (10% methanol and 5% acetic acid). The gels were washed in water 2–3 times and stored in water at room temperature until further analysis.

### 3.9. In-Gel Trypsin Digest from Silver-Stained Gels

Pooled tear samples were prepared by excising the bands from the silver-stained gels, which were first washed twice in ddH_2_O for 15 min. Bands of interest were cut out using a clean razor blade, chopped into 5–6 small pieces, and transferred to 1.5 mL Protein LoBind microcentrifuge tubes containing 100 µL of HPLC-grade water. After a 10 min incubation at room temperature with gentle vortexing, the gel pieces were destained using a destaining solution (0.4 g potassium ferricyanide (K_3_Fe(CN)_6_) in 200 mL sodium thiosulphate (0.2 g/L Na_2_S_2_O_3_•5H_2_O) until no bands were visible, then washed 4–5 times with Milli-Q H_2_O until transparent. The gel pieces were equilibrated in 100 µL of 50 mM ammonium bicarbonate buffer for 20 min, followed by two washes with 100 µL of 25 mM ammonium bicarbonate in 50% acetonitrile, each for 10 min, and a final wash in 100% acetonitrile for 10 min. The dehydrated gel slices were dried in a speed-vac for 5 min. For reduction and alkylation, the slices were covered with 10 mM DTT and incubated at room temperature for 30 min, then treated with an equal volume of 55 mM iodoacetamide for 45 min in the dark. The gel slices were washed with 25 mM ammonium bicarbonate for 10 min, dehydrated again with acetonitrile, and dried. Trypsin/Lys-C (20 ng/µL in 25 mM ammonium bicarbonate) was added to cover the gel slices (~30 µL), and the samples were incubated at 4 °C for 10–15 min before being covered with 25–30 µL of 25 mM ammonium bicarbonate for overnight digestion at 37 °C. To extract the tryptic peptides, 50 µL of acetonitrile was added, followed by 10 min of shaking at room temperature. The solution was transferred to a clean tube, and the gel slices were rehydrated with 30 µL HPLC-grade water for 10 min, followed by another extraction with 50 µL acetonitrile. The extracted solutions were combined, frozen at –80 °C, and lyophilized. The samples were cleaned using StageTips, reconstituted in 20 µL of Solution A (0.1% formic acid in HPLC water), and loaded onto Evosep tips for subsequent LC–MS/MS analysis.

### 3.10. Gene Ontology (GO) Analysis

GO analysis of the 1301 tear proteins was performed using the Database for Annotation, Visualization, and Integrated Discovery (DAVID) Bioinformatics software (https://david.ncifcrf.gov/ accessed on 18 August 2024) [42]. Only biological process and molecular function entries with *p*-value < 0.05 and % >1% were listed and plotted. The Proteomap was generated using Proteomaps software (https://www.proteomaps.net/ accessed on 8 September 2024).

## 4. Discussion and Conclusions

Tear film fluid presents an excellent sample resource for point-of-care testing (POCT) due to its non-invasive nature and ease of collection. Translation of tear film testing to the clinical environment, however, requires a thorough investigation of its composition through proteomics to identify novel biomarkers.

Based on our protein quantification, tear fluid samples have an average protein concentration of 10.3 ± 2.9 µg/µL, meaning that 0.5 µL of tear fluid contains approximately 5 µg of protein, which is sufficient for at least three technical replicates on an LC–MS/MS system. We observed that a further reduction of the sample volume to 0.25 µL led to a significant decrease in the number of proteins identified when analyzed with the in-capillary digestion protocol developed in this study (Figure S3). Therefore, 0.5 µL is considered the optimized volume for this in-capillary tear proteomics method.

This study developed a rapid and efficient in-capillary digestion workflow for tear proteomics that overcomes the challenges associated with small sample volumes and complex tear fluid composition. By utilizing only 0.5 µL of tear fluid, our method streamlines sample handling, minimizes sample loss and data errors, and enhances protein identification without the need for extensive desalting or fractionation. Although larger sample volumes may facilitate analytical procedures, they typically require more time to collect, which may induce reflex tearing. Therefore, developing a processing method that can be used to streamline analysis and provide improved results for the small volume of tear fluid samples represents a significant advancement in this field.

The optimized protocol detects an average of 816 proteins per sample, comparable to the standard Schirmer strip method, which typically identifies around 500–700 unique proteins [10,19,43,44,45,46,47]. Additionally, this protocol significantly outperforms the previous capillary-based studies, which identified only 100–200 proteins [8,27,28,29,30,31,32,33,34,35,36]. While our experimental workflow did detect some contamination, as evidenced by proteins identified in blank runs, the impact on results is minimal; only 20–30 proteins appeared in the blanks, and most were associated with a single peptide (Table S6). As a precaution, we recommend including one or more blank runs between the sample analyses to monitor contamination.

Our streamlined workflow coupled with LC–MS/MS-based bottom–up shotgun proteomics enables high-throughput tear proteomics investigations and biomarker discovery with improved sensitivity and efficiency. However, an intrinsic limitation of bottom–up shotgun proteomics using LC–MS/MS technology is that certain proteins, such as hydrophobic proteins or small proteins lacking suitable trypsin cleavage sites, may not be efficiently detected with this in-capillary digestion and LC–MS/MS workflow. In such cases, affinity-based targeted proteomics platforms, including Luminex (Luminex Corporation, Austin, TX, USA), Simoa technology (Quanterix Corporation, Billerica, MA, USA), PEA technology (Olink, Uppsala, Sweden)), and SomaScan (SomaLogic, Boulder, CO, USA), may offer viable alternatives [48]. Recent studies have explored this approach, with the Olink Target 96 Inflammation Panel used to evaluate tear proteomics pre-processing methods [3], and the Olink Explore 384 Inflammation Panel applied to identify tear biomarkers for keratoconus research [49]. These affinity-based platforms may have higher sensitivity for proteins less compatible with mass spectrometry; however, they often require larger sample volumes. Additionally, these targeted platforms are limited to pre-selected protein targets, potentially missing novel biomarkers that are not present on the panel. Moreover, compared to these targeted platforms, LC–MS/MS is generally more cost-effective, with per-sample costs of typically only a few dollars.

This innovative approach unlocks the potential for tear fluid as a non-invasive source of biomarkers, paving the way for advances in POCT diagnostics and personalized medicine in both ophthalmic and systemic diseases. The first point-of-care testing (POCT) devices for analyzing tear fluid use microfluidic collection tips, which are more similar to capillary collection [12,50,51]. Although LC–MS/MS requires specialized equipment, making it less suitable for clinical diagnostics, understanding the differences between Schirmer and capillary collection is essential. Conducting biomarker studies with collection methods that closely mimic POCT techniques can enhance the reliability of devices that bring this research into clinical practice.

In conclusion, our novel in-capillary trypsin digestion workflow significantly enhances tear proteomics by enabling the identification of 500–800 proteins from just 0.5 μL of tear fluid with minimal sample handling and a streamlined, scalable process. This efficient approach offers a high-throughput solution for biomarker discovery, addressing the limitations of low tear volume and complex composition, and positions tear fluid as a promising resource for clinical proteomics. Moving forward, our focus is on further improving this approach by incorporating Parallel Reaction Monitoring (PRM)-based targeted mass spectrometry technique to achieve a more precise quantitation of a number of potential biomarkers in tear fluid samples.

## Data Availability

The mass spectrometry proteomics data have been deposited to the ProteomeXchange Consortium via the PRIDE [52] partner repository with the dataset identifier PXD056978.

## Supplementary Materials

The following supporting information can be downloaded at: https://www.mdpi.com/article/10.3390/ijms252212239/s1.

## References

[1] Koduri M.A., Prasad D., Pingali T., Singh V.K., Shanbhag S.S., Basu S., Singh V. (2023). Optimization and evaluation of tear protein elution from Schirmer’s strips in dry eye disease. Indian J. Ophthalmol..

[2] Nattinen J., Aapola U., Jylha A., Vaajanen A., Uusitalo H. (2020). Comparison of Capillary and Schirmer Strip Tear Fluid Sampling Methods Using SWATH-MS Proteomics Approach. Transl. Vis. Sci. Technol..

[3] Vergouwen D.P.C., Schotting A.J., Endermann T., van de Werken H.J.G., Grashof D.G.B., Arumugam S., Nuijts R., Ten Berge J.C., Rothova A., Schreurs M.W.J. (2023). Evaluation of pre-processing methods for tear fluid proteomics using proximity extension assays. Sci. Rep..

[4] Gijs M., Arumugam S., van de Sande N., Webers C.A.B., Sethu S., Ghosh A., Shetty R., Vehof J., Nuijts R. (2023). Pre-analytical sample handling effects on tear fluid protein levels. Sci. Rep..

[5] Huang Z., Du C.X., Pan X.D. (2018). The use of in-strip digestion for fast proteomic analysis on tear fluid from dry eye patients. PLoS ONE.

[6] Chen X., Rao J., Zheng Z., Yu Y., Lou S., Liu L., He Q., Wu L., Sun X. (2019). Integrated Tear Proteome and Metabolome Reveal Panels of Inflammatory-Related Molecules via Key Regulatory Pathways in Dry Eye Syndrome. J. Proteome Res..

[7] Ananthi S., Venkatesh Prajna N., Lalitha P., Valarnila M., Dharmalingam K. (2013). Pathogen induced changes in the protein profile of human tears from Fusarium keratitis patients. PLoS ONE.

[8] Li N., Wang N., Zheng J., Liu X.M., Lever O.W., Erickson P.M., Li L. (2005). Characterization of human tear proteome using multiple proteomic analysis techniques. J. Proteome Res..

[9] Hagan S., Martin E., Enriquez-de-Salamanca A. (2016). Tear fluid biomarkers in ocular and systemic disease: Potential use for predictive, preventive and personalised medicine. EPMA J..

[10] Boerger M., Funke S., Leha A., Roser A.E., Wuestemann A.K., Maass F., Bahr M., Grus F., Lingor P. (2019). Proteomic analysis of tear fluid reveals disease-specific patterns in patients with Parkinson’s disease—A pilot study. Park. Relat. Disord..

[11] Rentka A., Koroskenyi K., Harsfalvi J., Szekanecz Z., Szucs G., Szodoray P., Kemeny-Beke A. (2017). Evaluation of commonly used tear sampling methods and their relevance in subsequent biochemical analysis. Ann. Clin. Biochem..

[12] Yang S.M., Lv S., Zhang W., Cui Y. (2022). Microfluidic Point-of-Care (POC) Devices in Early Diagnosis: A Review of Opportunities and Challenges. Sensors.

[13] Zhang S., Zeng J., Wang C., Feng L., Song Z., Zhao W., Wang Q., Liu C. (2021). The Application of Wearable Glucose Sensors in Point-of-Care Testing. Front. Bioeng. Biotechnol..

[14] Haghayegh F., Norouziazad A., Haghani E., Feygin A.A., Rahimi R.H., Ghavamabadi H.A., Sadighbayan D., Madhoun F., Papagelis M., Felfeli T. (2024). Revolutionary Point-of-Care Wearable Diagnostics for Early Disease Detection and Biomarker Discovery through Intelligent Technologies. Adv. Sci..

[15] Christodouleas D.C., Kaur B., Chorti P. (2018). From Point-of-Care Testing to eHealth Diagnostic Devices (eDiagnostics). ACS Cent. Sci..

[16] Murube J. (2009). Basal, reflex, and psycho-emotional tears. Ocul. Surf..

[17] Fullard R.J., Tucker D.L. (1991). Changes in human tear protein levels with progressively increasing stimulus. Investig. Ophthalmol. Vis. Sci..

[18] Sia R.K., Ryan D.S., Howard R.S., Haymes S., Zhou Y., Coe C.D., Bower K.S. (2016). Non-stimulated Tear Sample Collection Using Polyvinyl Alcohol (PVA) Foam and Polyester Wick. Int. J. Ophthalmol. Clin. Res..

[19] Jones G., Lee T.J., Glass J., Rountree G., Ulrich L., Estes A., Sezer M., Zhi W., Sharma S., Sharma A. (2022). Comparison of Different Mass Spectrometry Workflows for the Proteomic Analysis of Tear Fluid. Int. J. Mol. Sci..

[20] Tse J.S., Sze Y.H., Ka-Wai Cheung J., Li K.K., Lam T.C. (2023). A Protein Suspension-Trapping Sample Preparation for Tear Proteomics by Liquid Chromatography-Tandem Mass Spectrometry. J. Vis. Exp..

[21] Harkness B.M., Hegarty D.M., Saugstad J.A., Behrens H., Betz J., David L.L., Lapidus J.A., Chen S., Stutzman R., Chamberlain W. (2023). Experimental design considerations for studies of human tear proteins. Ocul. Surf..

[22] Bachhuber F., Huss A., Senel M., Tumani H. (2021). Diagnostic biomarkers in tear fluid: From sampling to preanalytical processing. Sci. Rep..

[23] Xiao J., Fu Y., Frenia K., Sikora J., Garwood K., Mental R., Labriola L.T. (2024). Tear fluid proteomic analysis with improved LC-MS/MS protocol. Investig. Ophthalmol. Vis. Sci..

[24] Posa A., Brauer L., Schicht M., Garreis F., Beileke S., Paulsen F. (2013). Schirmer strip vs. capillary tube method: Non-invasive methods of obtaining proteins from tear fluid. Ann. Anat..

[25] Tham M.L., Mahmud A., Abdullah M., Md Saleh R., Mohammad Razali A., Cheah Y.K., Mohd Taib N., Ho K.L., Mahmud M., Mohd Isa M. (2023). Tear Samples for Protein Extraction: Comparative Analysis of Schirmer’s Test Strip and Microcapillary Tube Methods. Cureus.

[26] Chang A.Y., Purt B. (2024). Biochemistry, Tear Film. StatPearls.

[27] Acera A., Vecino E., Rodriguez-Agirretxe I., Aloria K., Arizmendi J.M., Morales C., Duran J.A. (2011). Changes in tear protein profile in keratoconus disease. Eye.

[28] Soria J., Duran J.A., Etxebarria J., Merayo J., Gonzalez N., Reigada R., Garcia I., Acera A., Suarez T. (2013). Tear proteome and protein network analyses reveal a novel pentamarker panel for tear film characterization in dry eye and meibomian gland dysfunction. J. Proteom..

[29] Ananthi S., Chitra T., Bini R., Prajna N.V., Lalitha P., Dharmalingam K. (2008). Comparative analysis of the tear protein profile in mycotic keratitis patients. Mol. Vis..

[30] Csosz E., Boross P., Csutak A., Berta A., Toth F., Poliska S., Torok Z., Tozser J. (2012). Quantitative analysis of proteins in the tear fluid of patients with diabetic retinopathy. J. Proteom..

[31] Kallo G., Emri M., Varga Z., Ujhelyi B., Tozser J., Csutak A., Csosz E. (2016). Changes in the Chemical Barrier Composition of Tears in Alzheimer’s Disease Reveal Potential Tear Diagnostic Biomarkers. PLoS ONE.

[32] Salvisberg C., Tajouri N., Hainard A., Burkhard P.R., Lalive P.H., Turck N. (2014). Exploring the human tear fluid: Discovery of new biomarkers in multiple sclerosis. Proteom. Clin. Appl..

[33] Yenihayat F., Altintas O., Kasap M., Akpinar G., Guzel N., Celik O.S. (2018). Comparative proteome analysis of the tear samples in patients with low-grade keratoconus. Int. Ophthalmol..

[34] Ponzini E., Santambrogio C., De Palma A., Mauri P., Tavazzi S., Grandori R. (2022). Mass spectrometry-based tear proteomics for noninvasive biomarker discovery. Mass Spectrom. Rev..

[35] Zhan X., Li J., Guo Y., Golubnitschaja O. (2021). Mass spectrometry analysis of human tear fluid biomarkers specific for ocular and systemic diseases in the context of 3P medicine. EPMA J..

[36] Green-Church K.B., Nichols K.K., Kleinholz N.M., Zhang L., Nichols J.J. (2008). Investigation of the human tear film proteome using multiple proteomic approaches. Mol. Vis..

[37] Aydin E., Dhar P., Gokhale M., Chong L., Azizoglu S., Suphioglu C. (2022). A Review of Emerging Tear Proteomics Research on the Ocular Surface in Ocular Allergy. Biology.

[38] Olson B.J., Markwell J. (2007). Assays for determination of protein concentration. Curr. Protoc. Protein Sci..

[39] Kielkopf C.L., Bauer W., Urbatsch I.L. (2020). Methods for Measuring the Concentrations of Proteins. Cold Spring Harb. Protoc..

[40] Pieczynski J., Szulc U., Harazna J., Szulc A., Kiewisz J. (2021). Tear fluid collection methods: Review of current techniques. Eur. J. Ophthalmol..

[41] Krajcikova K., Glinska G., Tomeckova V. (2022). Effect of tear fluid sampling and processing on total protein quantity and electrophoretic pattern. Taiwan J. Ophthalmol..

[42] Sherman B.T., Hao M., Qiu J., Jiao X., Baseler M.W., Lane H.C., Imamichi T., Chang W. (2022). DAVID: A web server for functional enrichment analysis and functional annotation of gene lists (2021 update). Nucleic Acids Res..

[43] Lopez-Lopez M., Regueiro U., Bravo S.B., Chantada-Vazquez M.D.P., Varela-Fernandez R., Avila-Gomez P., Hervella P., Lema I. (2021). Tear Proteomics in Keratoconus: A Quantitative SWATH-MS Analysis. Investig. Ophthalmol. Vis. Sci..

[44] Vaajanen A., Nattinen J., Aapola U., Gielen F., Uusitalo H. (2021). The effect of successful trabeculectomy on the ocular surface and tear proteomics—A prospective cohort study with 1-year follow-up. Acta Ophthalmol..

[45] O’Leary O.E., Schoetzau A., Amruthalingam L., Geber-Hollbach N., Plattner K., Jenoe P., Schmidt A., Ullmer C., Drawnel F.M., Fauser S. (2020). Tear Proteomic Predictive Biomarker Model for Ocular Graft Versus Host Disease Classification. Transl. Vis. Sci. Technol..

[46] Pieragostino D., Lanuti P., Cicalini I., Cufaro M.C., Ciccocioppo F., Ronci M., Simeone P., Onofrj M., van der Pol E., Fontana A. (2019). Proteomics characterization of extracellular vesicles sorted by flow cytometry reveals a disease-specific molecular cross-talk from cerebrospinal fluid and tears in multiple sclerosis. J. Proteom..

[47] Gerber-Hollbach N., Plattner K., O’Leary O.E., Jenoe P., Moes S., Drexler B., Schoetzau A., Halter J.P., Goldblum D. (2018). Tear Film Proteomics Reveal Important Differences Between Patients With and Without Ocular GvHD After Allogeneic Hematopoietic Cell Transplantation. Investig. Ophthalmol. Vis. Sci..

[48] Cui M., Cheng C., Zhang L. (2022). High-throughput proteomics: A methodological mini-review. Lab. Investig..

[49] Gijs M., Vergouwen D., Visser N., Dickman M., Ollivier R., Berendschot T.T., Nuijts R. (2023). Using the Olink proteomics tear fluid biomarker approache to better understand keratoconus. Investig. Ophthalmol. Vis. Sci..

[50] Sachdeva S., Davis R.W., Saha A.K. (2020). Microfluidic Point-of-Care Testing: Commercial Landscape and Future Directions. Front. Bioeng. Biotechnol..

[51] TearLab Collection Guide. https://www.labtician.com/therapeutics/wp-content/uploads/2020/04/TearLab-Basic-Troubleshooting-Collection-Guide-FINAL-8-11-16.pdf.

[52] Perez-Riverol Y., Bai J., Bandla C., Garcia-Seisdedos D., Hewapathirana S., Kamatchinathan S., Kundu D.J., Prakash A., Frericks-Zipper A., Eisenacher M. (2022). The PRIDE database resources in 2022: A hub for mass spectrometry-based proteomics evidences. Nucleic Acids Res.

