# Supplementary material for: High-Throughput Tear Proteomics via In-Capillary Digestion for Biomarker Discovery
Source: Int J Mol Sci. 2024 Nov 14;25(22):12239. doi: 10.3390/ijms252212239 (PMC11594680; doi:10.3390/ijms252212239)
Supplement: Supplementary file 1 [file ijms-25-12239-s001.zip › ijms-3271741-supplementary.pdf]

Figure S1

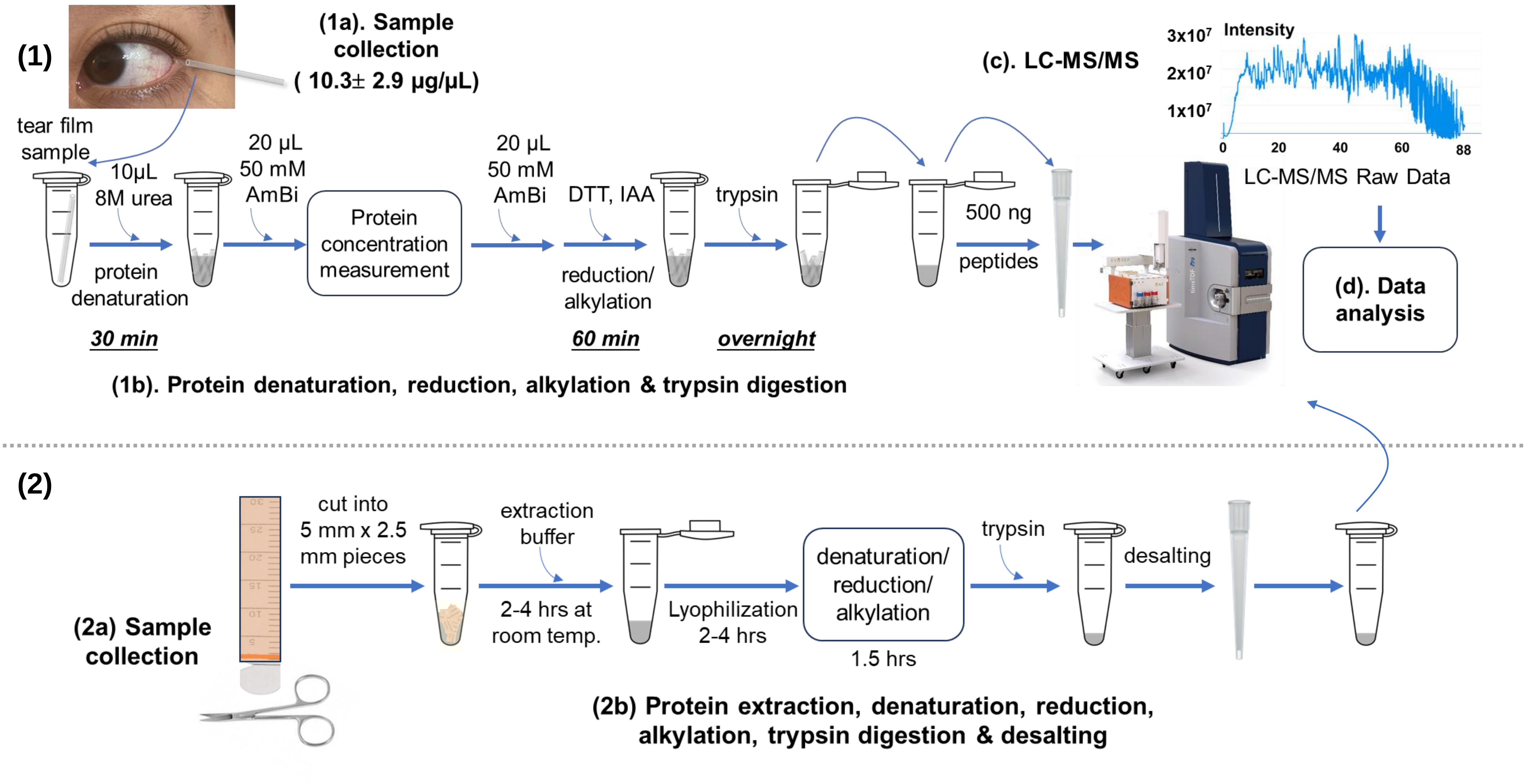

(1)
(1a). Sample collection
(10.3± 2.9 µg/µL)
tear film sample
10µL
8M urea
protein denaturation
30 min
20 µL
50 mM
AmBi
Protein concentration measurement
20 µL
50 mM
AmBi
DTT, IAA
reduction/ alkylation
60 min
trypsin
overnight
500 ng
peptides
(1b). Protein denaturation, reduction, alkylation & trypsin digestion
(c). LC-MS/MS
3x10^7
2x10^7
1x10^7
Intensity
0
20
40
60
88
LC-MS/MS Raw Data
(d). Data analysis
(2)
(2a) Sample collection
cut into
5 mm x 2.5
mm pieces
extraction buffer
2-4 hrs at room temp.
Lyophilization
2-4 hrs
denaturation/ reduction/ alkylation
1.5 hrs
trypsin
desalting
(2b) Protein extraction, denaturation, reduction, alkylation, trypsin digestion & desalting

Figure S1. Novel In-Capillary Digestion Workflow for Enhanced Tear Proteomics in Biomarker Discovery.

(1) The in-capillary digestion workflow developed in this study was optimized for tear proteomics using minimal sample volumes of 0.5 µL, collected with glass capillary tubes. **(1a)** Tear fluid (0.5 µL) was collected in glass capillary tubes, with an average protein concentration of 10.3 $\pm$ 2.9 µg/µL. **(1b)** The experimental workflow includes steps for protein denaturation, reduction, alkylation, and trypsin digestion. After collection, the capillary tube containing the tear sample was placed in a Protein LoBind microcentrifuge tube and stored at 4° C or on dry ice for transport. Without removing the tear sample from the capillary, 50 µL of 8 M urea solution was added to the tube, which was then vigorously vortexed and centrifuged at ~20,000 x g in a benchtop microcentrifuge for three cycles, breaking the capillary into fragments to ensure thorough mixing. The sample was incubated at room temperature for 30 minutes to achieve protein denaturation. Subsequently, 20 µL of 50 mM ammonium bicarbonate solution was added to dilute the urea concentration to 2.67 M, and protein concentration was measured via A280 absorbance on a NanoDrop spectrophotometer. After quantification, additional 50 mM ammonium bicarbonate solution was added to prepare for DTT reduction, IAA alkylation, and trypsin digestion. Once digestion was complete, and the pH adjusted to 2–3, 500 ng of digested peptides were directly loaded onto an Evosep tip for LC-MS/MS analysis **(c)**, followed by data processing and analysis **(d)**.

(2) Comparative workflow for tear proteomics using the traditional Schirmer strip collection method. **(2a)** Tear samples were collected using Schirmer strips, which were then cut into 5 mm x 2.5 mm pieces and placed into a Protein LoBind microcentrifuge tube. A protein extraction buffer was added to cover the Schirmer strip pieces, and the mixture was incubated at room temperature for 2–4 hours with shaking. **(2b)** Following incubation, the sample was centrifuged, and the strip pieces were removed. The extracted protein solution was then lyophilized, followed by denaturation with 8 M urea, reduction, and alkylation, and overnight trypsin digestion. The tryptic peptides were desalted before LC-MS/MS **(c)** and subsequent data analysis **(d)**.

Figure S2

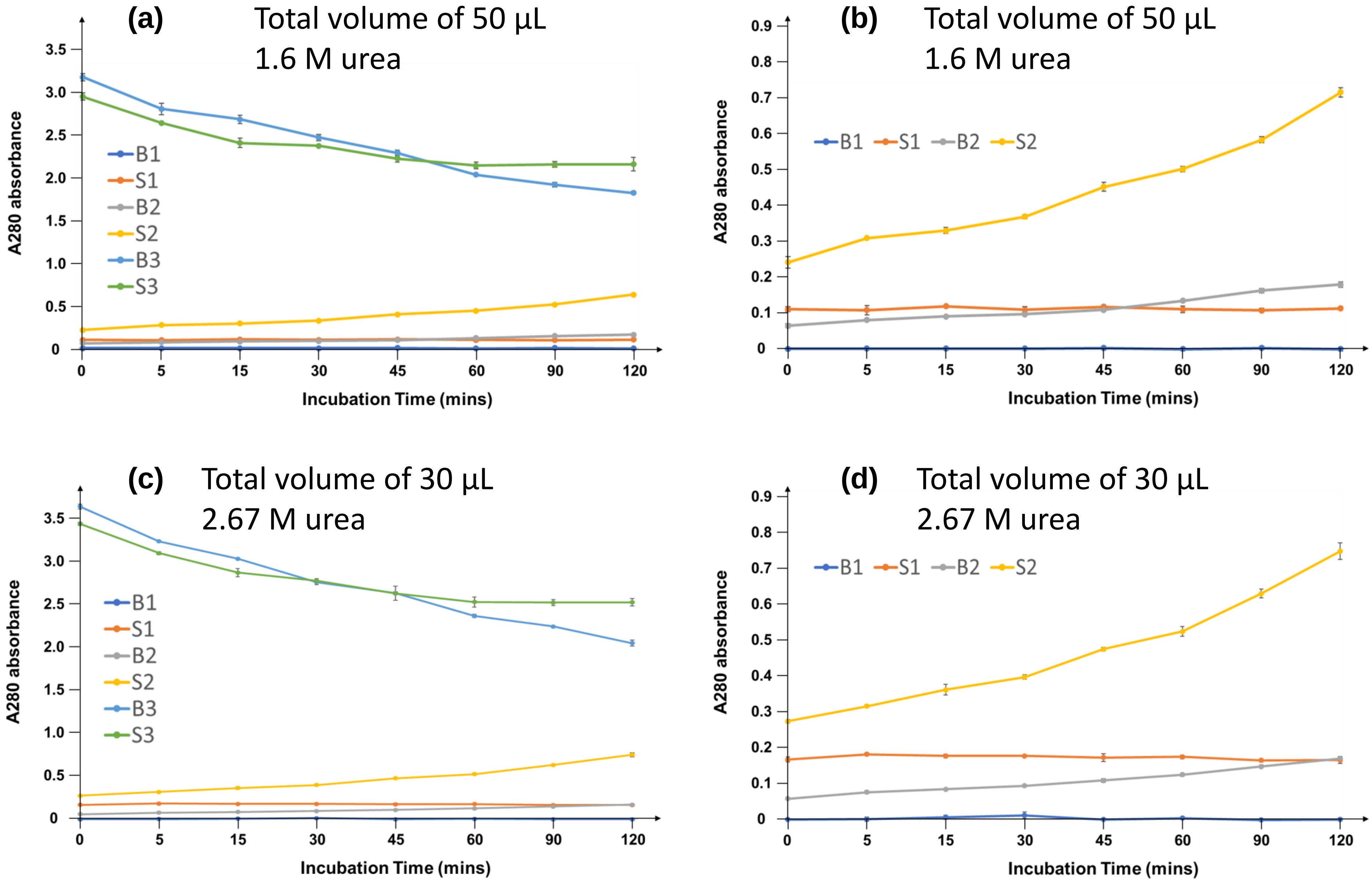

B1. Buffer without DTT & IAA
B2. Buffer with10 mM DTT
B3. Buffer with10 mM DTT, 25 mM IAA

S1. Tear sample without DTT & IAA
S2. Tear sample with 10 mM DTT
S3. Tear samples with10 mM DTT, 25 mM IAA

Figure S2. The effect of dithiothreitol (DTT) and iodoacetamide (IAA) on tear protein concentration measurements.

Pooled tear samples were aliquoted into capillary tubes, each containing 0.5 µL of tear fluid, and placed in individual microcentrifuge tubes. To each tube, 10 µL of 8 M urea solution was added, and the samples were incubated at room temperature for 30 minutes to denature the proteins. Next, either 40 µL (**a & b**) or 20 µL (**c & d**) of 50 mM ammonium bicarbonate solution was added to dilute the urea concentration. DTT and IAA were then added to the tubes as follows -- S1: No DTT or IAA added; S2: 10 mM DTT added; S3: 10 mM DTT added followed by 25 mM IAA. The volumes in all tubes were adjusted to be equal using 50 mM ammonium bicarbonate solution. Corresponding buffer controls (B1, B2, and B3) were prepared without tear proteins to match the experimental conditions of the tear samples. The tear protein and buffer solutions were incubated at room temperature, and the A280 absorbance was measured at 0, 5, 15, 30, 45, 60, 90, and 120 minutes using a NanoDrop spectrophotometer. **(a)** A280 absorbance readings for B1, B2, B3, S1, S2, and S3 were taken at various time points after 40 µL of 50 mM ammonium bicarbonate was added, diluting the urea concentration from 8 M to 1.6 M prior to the addition of DTT and IAA, or an equivalent volume of 50 mM ammonium bicarbonate solution. **(b)** A280 absorbance readings for B1, B2, S1, and S2 are shown to visualize the data for the conditions described in (a). **(c)** A280 absorbance readings for B1, B2, B3, S1, S2, and S3 were taken at various time points after 20 µL of 50 mM ammonium bicarbonate was added, diluting the urea concentration from 8 M to 2.67 M before the addition of DTT and IAA, or an equivalent volume of 50 mM ammonium bicarbonate solution. **(d)** A280 absorbance readings for B1, B2, S1, and S2 are shown to visualize the data for the conditions described in (**c**).

Figure S3

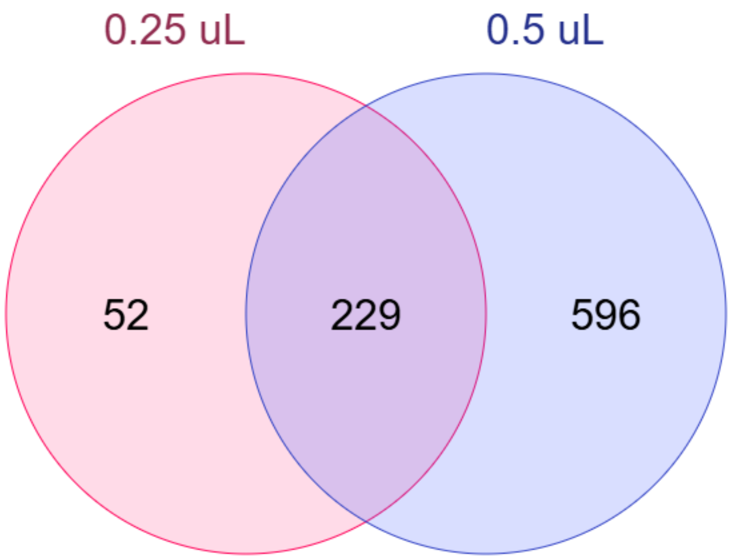

Figure S3. Comparison of Tear Proteomes Identified Using 0.25 μL vs. 0.5 μL of Tear Fluid Samples.

This figure demonstrates the difference in protein identification between using 0.25 μL and 0.5 μL of tear fluid in the in-capillary digestion workflow developed in this study. A total of 825 proteins were identified with 0.5 μL of tear fluid, compared to 281 proteins detected with 0.25 μL, highlighting the improved protein yield and sensitivity achieved with the optimized 0.5 μL sample volume.
